# Phonon and heat transport control using pillar-based phononic crystals

**DOI:** 10.1080/14686996.2018.1542524

**Published:** 2018-11-01

**Authors:** Roman Anufriev, Masahiro Nomura

**Affiliations:** a Institute of Industrial Science, The University of Tokyo, Tokyo, Japan; b PRESTO, Japan Science and Technology Agency, Saitama, Japan

**Keywords:** Phononic crystals, thermoelectrics, silicon, thermal conductivity, energy harvesting, 10 Engineering and Structural materials, 210 Thermoelectronics / Thermal transport / Insulators

## Abstract

Phononic crystals have been studied for the past decades as a tool to control the propagation of acoustic and mechanical waves. Recently, researchers proposed that nanosized phononic crystals can also control heat conduction and improve the thermoelectric efficiency of silicon by phonon dispersion engineering. In this review, we focus on recent theoretical and experimental advances in phonon and thermal transport engineering using pillar-based phononic crystals. First, we explain the principles of the phonon dispersion engineering and summarize early proof-of-concept experiments. Next, we review recent simulations of thermal transport in pillar-based phononic crystals and seek to uncover the origin of the observed reduction in the thermal conductivity. Finally, we discuss first experimental attempts to observe the predicted thermal conductivity reduction and suggest the directions for future research.

## GRAPHICAL ABSTRACT

**Figure:**
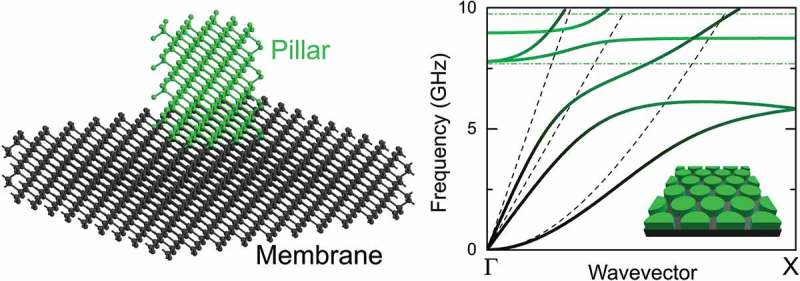

## 1. Introduction

Phononic crystals (PnCs) are artificial periodic structures that control the propagation of phonons—quanta of lattice vibrations—using the effect of phonon interference caused by the inherent wave nature of phonons [1–3]. In semiconductors, where phonons are the primary carriers of heat, such control over phonon transport promises to revolutionize the thermal management. The concept of PnCs offers thermal diodes, cloaks, guides, insulators and other devices that can improve both heat dissipation and thermal insulation in microelectronics [2–4]. Moreover, PnCs can reduce the thermal conductivity [5,6]. Thus, PnCs can enhance the thermoelectric efficiency of poor thermoelectric materials, such as silicon [7–9], and be used in energy harvesting applications [10,11] along with other nanostructured [11–14] and hierarchical [15,16] materials.

Historically, planar PnCs were mainly hole-based, that is consisted of thin plates with periodic arrays of holes. The periodic hole boundaries systematically reflect phonons thus stimulating phonon interference, which changes phonon dispersion relation [2]. A comprehensive review on phonon dispersion engineering for acoustic waves manipulations using PnCs was provided by Pennec et al. [17] in 2010. Since then, many studies demonstrated that hole-based PnCs could also reduce the thermal conductivity [18], though the mechanisms of this reduction proved to be different from the expected phonon interference [19–24]. However, the hole-based PnCs have a drawback as a thermoelectric material: although holes reduce the thermal conductivity (κ) by phonon scattering, dense arrays of holes also scatter the electrons, thus reducing the electrical conductivity (σ). This reduction in the electrical conductivity limits the gain in thermoelectric figure of merit *ZT = *σ*S* ^2^ *T/*κ, where *S* is the Seebeck coefficient [23]. Moreover, holes reduce the material volume and thus limit thermoelectric power generation. To overcome these limitations, PnCs should not affect the interior of the material but act externally.

A decade ago, Pennec et al. [25] and Wu et al. [26] proposed that the mechanical properties of a thin plate can be changed by depositing external periodic pillars on the surface of the plate, thus forming the pillar-based PnC. Since then, many theoretical and experimental works studied pillar-based PnCs and their applications in mechanical and thermal engineering. During this time, the simulation methods spanned from finite element method (FEM) to molecular dynamics simulations, while experiments ranged from transmittance measurements to Brillouin light scattering and time-domain thermoreflectance techniques.

Here, we review the past decade of research on sound and heat control using pillar-based PnCs. First, we explain the principles of phonon dispersion engineering using pillar-based PnCs (Section 2). Next, we show how the changes in phonon dispersion were verified experimentally (Section 3). Finally, we review recent simulations predicting a reduction of the thermal conductivity by the pillar-based PnCs (Section 4) and experimental attempts to verify these predictions (Section 5).

## 2. Physics of local resonances and phononic bandgaps

**Figure 1.**
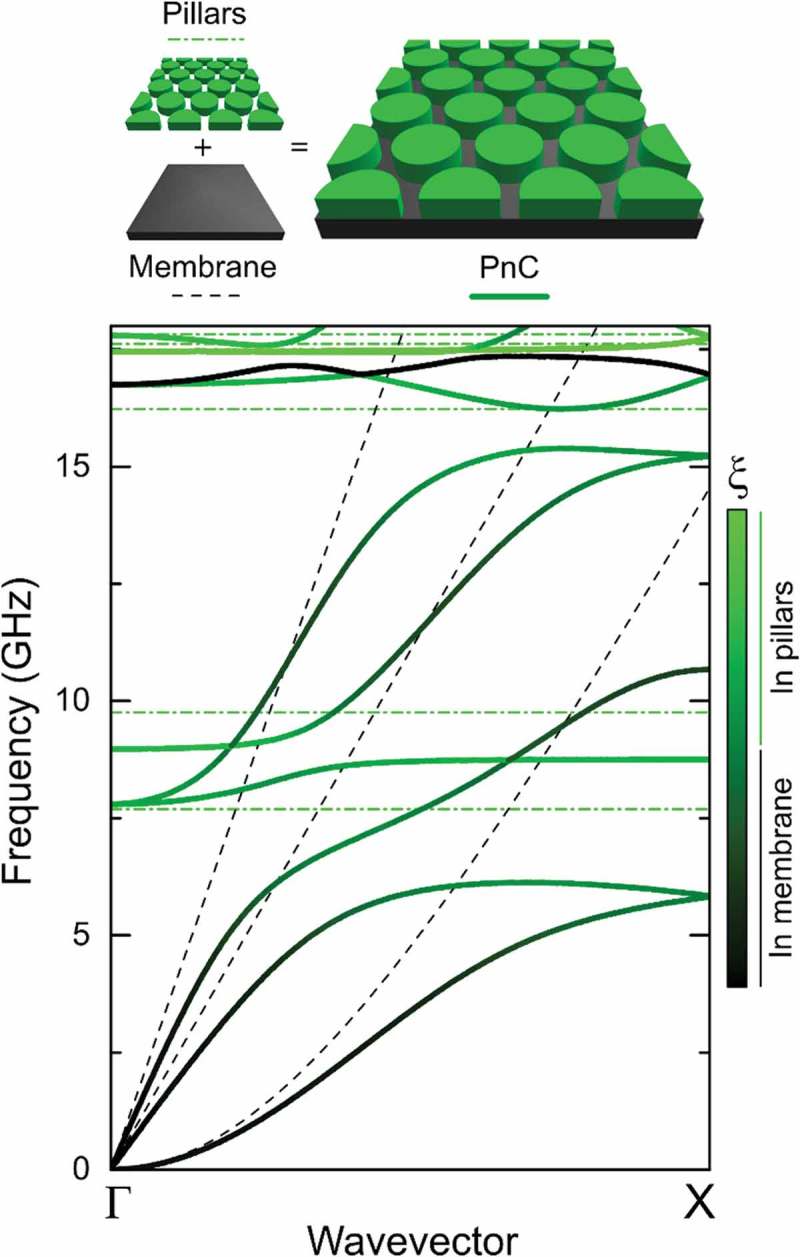
Phonon dispersion of a pillar-based PnC (solid lines), a silicon membrane (dashed lines), and resonant frequencies of a pillar (horizontal dash-dotted lines). The color of the branches shows the physical location of the modes (ξ). Details of the simulated structure can be found in Ref [30].

Let us consider the two mechanisms that change the propagation characteristics of elastic waves in pillar-based PnCs. First, like in the hole-based PnCs [2], the periodicity of pillars creates Bragg diffraction of mechanical waves [27], similar to that of photons in photonic crystals [28,29]. For example, the first two branches in Figure 1 are flattened due to the periodicity of the pillars [30]. Second, unlike holes, pillars introduce additional flat branches in the phonon dispersion. Figure 1 shows how the pillars change the original membrane modes (dashed lines) so that the branches become flattened (solid lines) near the eigenfrequencies of the pillars (horizontal dash-dotted lines) [30]. This mode flattening is often described as hybridization between the membrane modes and the resonant frequencies of the pillars. Analyzing the relative height of the mode location (ξ) [30,31], we find that the states corresponding to the flattened regions are localized inside the pillars (green color) [30,32–34]. Such localized states at the resonant frequencies are called local resonances.

The flattening of the dispersion branches due to both Bragg diffraction and the local resonances can form phononic bandgaps—the ranges of frequency, in which phonons cannot propagate [27,35,36]. The frequency of the Bragg bandgap is linked to the period of the PnCs and thus affects only phonons with the wavelengths of about the characteristic size of the system [27,35,37]. The frequencies of the local resonance bandgaps are linked to the resonant frequencies of the pillars [27], which can be tuned via structural parameters, such as pillar diameter [30,38] and height [25,26,30,38,39], or material parameters, such as density and Young modulus [25,30]. Thus, the local resonance bandgaps can be opened at higher [27] or lower [39,40] frequencies than the Bragg bandgap. Moreover, unlike Bragg bandgaps, the local resonance bandgaps are independent of the periodicity of pillars and are insensitive to the period disorder [27,39].

Together, the Bragg and local resonance bandgaps can cover a broad range of frequencies, which makes pillar-based PnCs ideal for waveguiding [37,41–43]. To further broaden the frequency range, researchers proposed the hybrid hole/pillar-based PnCs [44,45]. Such hybrid PnCs take advantages of both wide Bragg gaps of hole-based PnCs and multiple high-frequency bandgaps of pillar-based PnCs.

## 3. Measurements of phononic bandgaps and phonon dispersion

**Figure 2.**
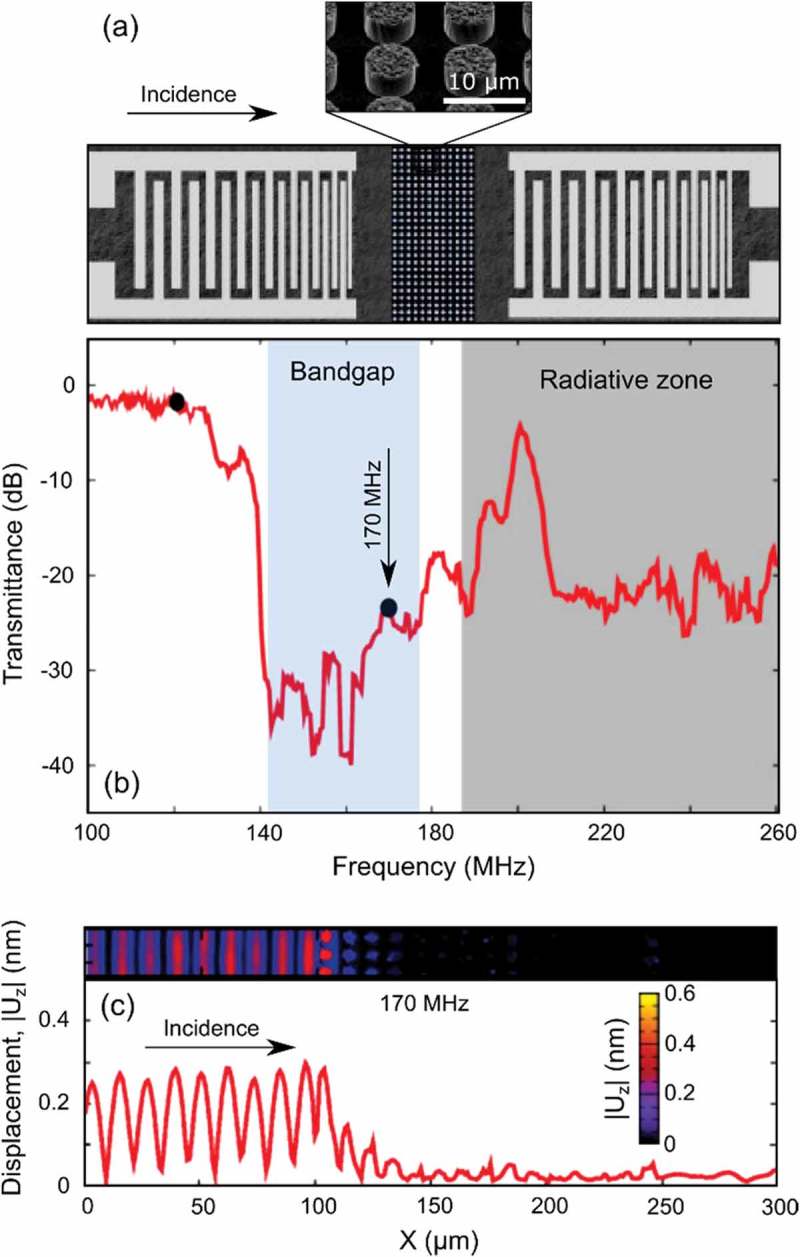
(a) Schematic of the sample used by Achaoui et al. [36] to study the phonon transmission through an array of nickel pillars of 6.4 µm in diameter and 4.7 µm in height. (b) Transmittance spectrum shows a phononic bandgap. (c) The out-of-plane displacement map shows the reflection of 170 MHz phonons from the PnC region. Reprinted figure with permission from [36]. Copyright 2018 by the American physical Society.

Early experimental works on pillar-based PnCs focused on measuring transmittance spectra, in which the phononic bandgaps appeared as dips. Experiments demonstrated the formation of both Bragg and local resonance bandgaps for surface acoustic waves due to the metallic pillars on the surface of bulk materials [26,36,39,46]. To give an example, Figure 2(a) shows the experiment by Achaoui et al. [36], who measured the transmittance through an array of nickel pillars on a lithium niobate substrate. The measured transmittance spectrum has a dip corresponding to the phononic bandgap (Figure 2(b)). The displacement map (Figure 2(c)) shows that the array of pillars reflects phonons with the frequency inside the bandgap.

However, the transmittance spectra could not provide much information about other changes of phonon dispersion. To measure the phonon dispersion directly, Trzaskowska et al. [47] applied the Brillouin light scattering technique and demonstrated that nickel pillars, deposited on a silicon substrate, strongly flatten the dispersion relation for the surface acoustic wave at the frequencies below 6 GHz. Later Brillouin light scattering experiments [31,48] demonstrated that pillars made of aluminum or gold could flatten phonon dispersion at even higher frequencies (up to 30 GHz), similarly to the hole-based PnCs.

**Figure 3.**
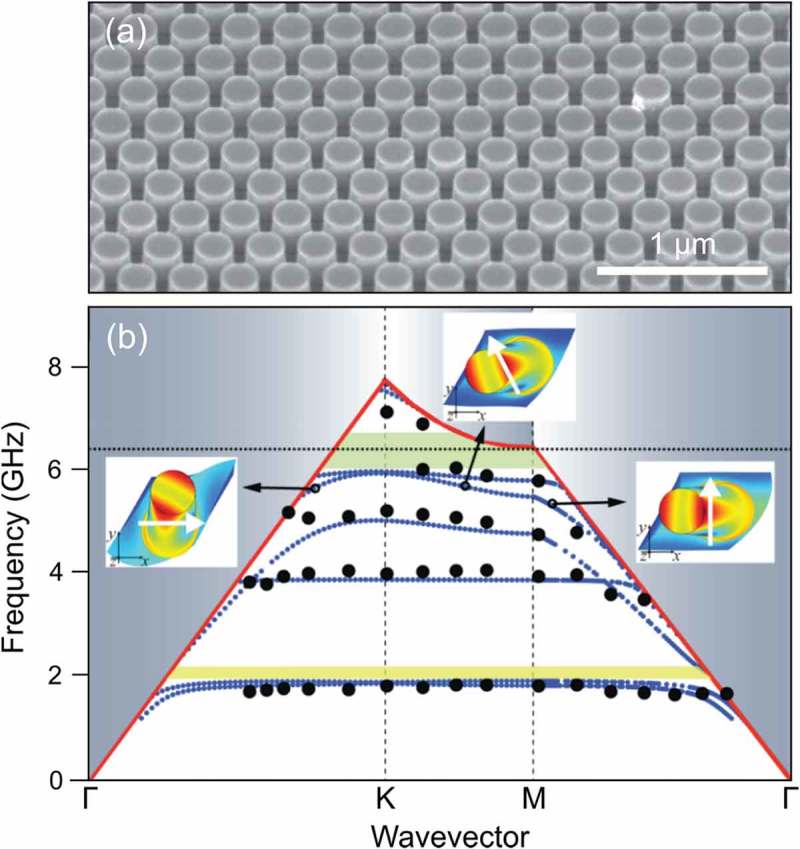
(a) Lithium niobate pillar-based PnC with pillars of 240 nm in diameter and 225 nm in height [49]. (b) Phonon dispersion of a PnC measured using Brillouin light scattering technique. The experimental data (black dots) agree with the result of the FEM simulations (small blue dots). Reprinted figure with permission from [49]. Copyright 2018 by the American Physical Society.

To enhance the hybridization of the pillar and substrate modes, Yudistira et al. [49] used etching through a chromium mask to create a monolithic PnC. Figure 3 shows this PnC and its phonon dispersion measured using the Brillouin light scattering technique [49]. The experimental data are consistent with the predictions of FEM simulations. The simulation also indicates that the observed modes are localized in the pillars, as shown in insets of Figure 3(b).

Moreover, using the Brillouin light scattering, Graczykowski et al. [50] studied mode propagation parallel and perpendicular to silicon strips on a silicon substrate, whereas Kargar et al. [51] observed localized states in free-standing GaAs nanowires.

## 4. Simulations of heat conduction

The phononic bandgaps, considered so far, are not the only result of phonon interference in PnCs. Also, the interference can flatten phonon dispersion across the entire phonon spectrum, including the frequencies much higher than the bandgaps [6,52]. The flattened branches of phonon dispersion imply the reduced group velocity and modified density of states [6,52–54]. Thus, the phonon interference can reduce the thermal conductivity of the structure and improve its thermoelectric efficiency.

In 2014, Davis and Hussein [55] proposed to use pillar-based PnCs to suppress heat conduction for thermoelectric applications. Using the lattice dynamics simulations, they demonstrated a 50% reduction in thermal conductivity of silicon membranes by adding periodic nanopillars of a few nanometers in size on the surface. Wei et al. [33,56] used molecular dynamics simulation to study the dependence of this effect on the geometrical parameters of the system. They confirmed that nanopillars could reduce the thermal conductivity by 50% and demonstrated that this reduction depends on the pillar diameter and the ratio of the pillar volume to that on the membrane within a unit cell [33]. This conclusion was confirmed by Honarvar et al. [57].

**Figure 4.**
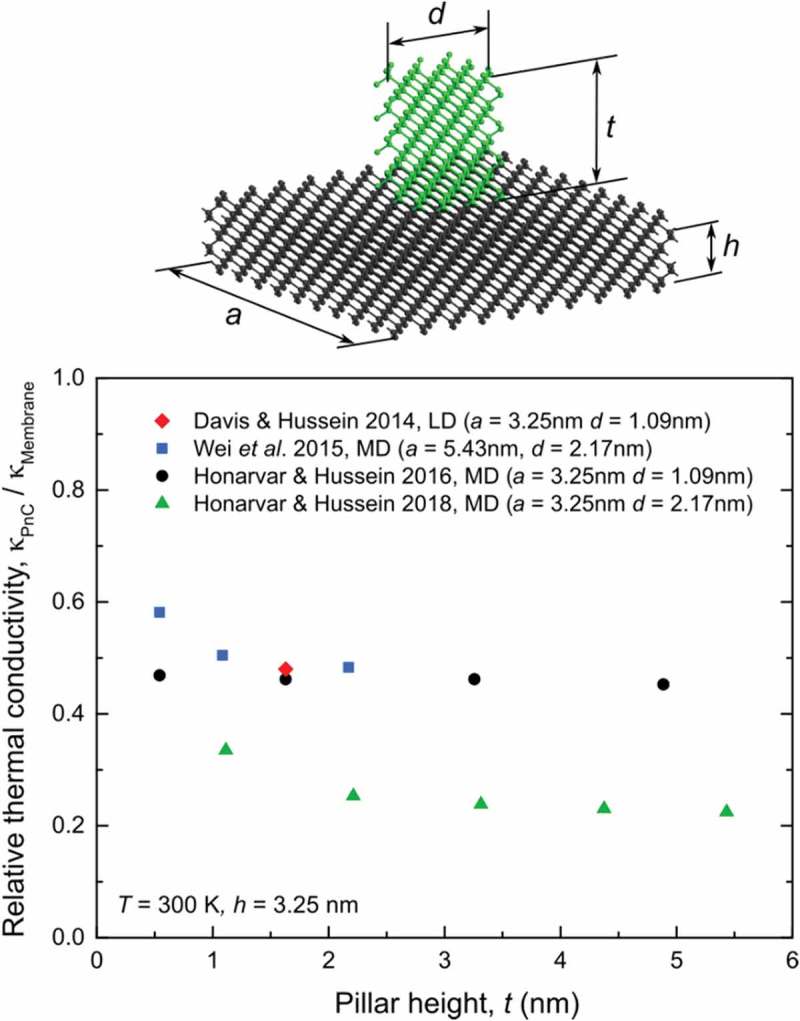
Example of a molecular dynamics model of a pillar-based PnC and the dependence of the thermal conductivity normalized by that of a membrane on the pillar height (*t*) found in Refs [33,34,55,58].

Moreover, Wei et al. [33] found that the reduction in thermal conductivity is almost independent of the pillar height, which was also confirmed by Honarvar and Hussein [58]. Figure 4 gathers the literature data on the thermal conductivity dependence on the pillar height. The data show that the pillar height (*t*) has no significant impact, at least for the pillars taller than several angstroms, Recently, Honarvar and Hussein [34] studied the height dependence in more details. They showed that even nanopillars of a few angstroms in height already reduce the thermal conductivity by more than 50%, but as nanopillars become taller this reduction gradually saturates.

The dependence of the thermal conductivity reduction on the pillar height is the key to uncovering the origin of this reduction. Let us look at this it more detail. Usually, researchers tentatively attribute the thermal conductivity reduction to the impact of local resonances on the phonon dispersion [32–34,55,57,58]. Indeed, the occurrence of the local resonances was demonstrated by different simulation techniques [30,38,55,58], and since the local resonances flatten the dispersion branches (Figure 1), the average group velocity becomes lower [32,33,57]. Hence, this effect can be linked to the thermal conductivity reduction. However, as we noted in our recent work [30], producing additional resonant states, with however low group velocity, does not necessarily reduce the thermal conductivity because these new states increase the density of states and thus may, on the contrary, enhance heat conduction.

In general, the thermal conductivity of pillar-based PnCs is affected by both the local resonances and the phonon interference due to the pillar periodicity, similar to that in hole-based PnCs [52,59]. One way to distinguish these two effects is to change the pillar height. As the height increases, the impact of periodicity remains almost the same but the impact of local resonances changes because the density of local resonances grows proportionally to the pillar height [30]. Thus, if the reduction in thermal conductivity is caused by the local resonances, this reduction should become stronger as pillars become taller. However, the literature data (Figure 4) show that the pillar height has only a minor impact on the thermal conductivity, whereas the main impact comes from the mere addition of the pillars, i.e. from the introduction of periodicity. Even monolayer-high pillars, which have almost no local resonances in the relevant frequency range, already reduce the thermal conductivity by 50%, as was demonstrated in our recent work [30] and can also be seen in Ref [58]. and its supplementary materials. These facts suggest that the reduction in thermal conductivity observed in simulations is caused mainly by the periodicity of the pillars rather than by local resonances.^1^

However, the nanopillars as small as a few nanometers may also act as surface roughness and decrease the thermal conductivity by the diffuse phonon scattering [60] instead of phonon interference effects. Xiong et al. [32] demonstrated that nanopillars not only generate local resonances but also scatter the phonons and reduce their mean free path by one order of magnitude. Similarly, Neogi et al. [61] concluded that the reduction in thermal conductivity is mainly caused by the diffuse scattering of phonons on nanopillars whereas local resonances play only a minor role. Recently, Honarvar and Hussein [34] too found that nanopillars shorten both phonon mean free path and lifetimes by the diffuse scattering, but argued that local resonances still play the leading role in the thermal conductivity reduction.

## 5. Experimental measurements of the thermal properties

**Figure 5.**
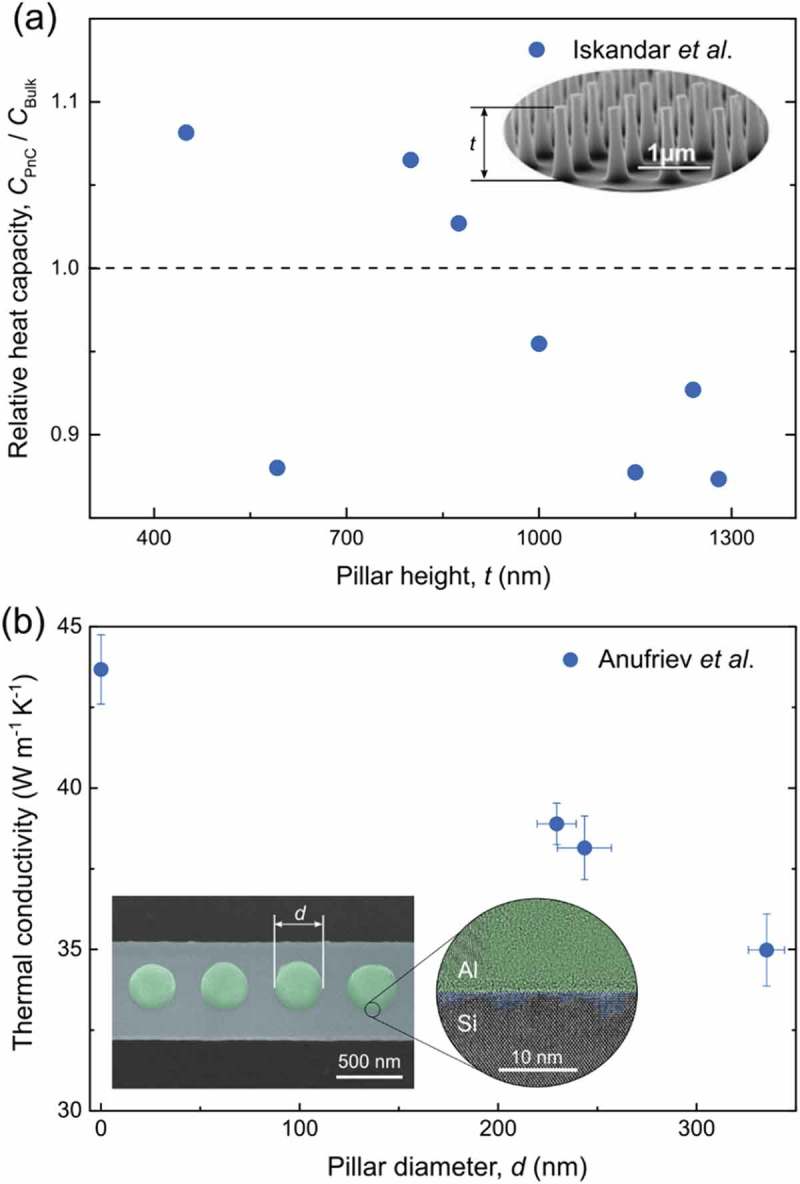
(a) Relative heat capacity of bulk silicon samples with pillars on the surface measured by Iskandar et al. [62] at room temperature. The inset shows one of the samples (the shape and size are different for every sample). Reprinted from [62], with the permission of AIP Publishing. (b) The thermal conductivity of the nanobeams with pillars of different diameters measured by Anufriev et al. [63] at room temperature. The inset shows the fragment of the nanobeam and transmission electron microscopy image of the pillar/beam interface featuring surface roughness.

Whereas the literature on heat conduction in pillar-based PnCs is rich of theoretical predictions and simulations of different types, only a few experimental works tried to verify these predictions yet. Iskandar et al. [62] studied thermal properties of bulk silicon with arrays of periodic pillars on the surface. Using the reactive ion etching, they created eight samples with different dimensions of the pillars and measured changes in the heat capacity of these samples as compared to the original silicon wafer. In Figure 5(a), we replot their room-temperature data as a function of the pillar height (although pillar shape and diameter also differ for each sample). The measured heat capacity values are within ±13% of the bulk value and show no apparent dependence on the pillar height. Nor does the heat capacity seem to correlate with any other geometrical parameter of the pillars. Iskandar et al. [62] attributed the observed changes in heat capacity to the impact of local resonance. However, the existing theoretical literature cannot explain why some samples would show an increase whereas other would show a decrease in the heat capacity. Moreover, the measured temperature dependence does not reveal any significant changes of the relative heat capacity, although theoretically one may expect a stronger impact of the phonon interference at low temperatures [18] as phonon wavelengths and mean free paths become longer.

To test the theoretical predictions more directly, Anufriev et al. [63] measured the thermal conductivity of thin silicon beams with aluminum pillars of different diameters. Figure 5(b) shows that the pillars indeed reduce the thermal conductivity, and this reduction is stronger for the pillars of larger diameter. However, contrary to the theoretical predictions [30,63], the temperature dependent measurements did not reveal significant changes in the thermal conductivity reduction at low temperatures. Analyzing the interface between the pillars and the beams, they found that the deposition of aluminum pillars causes amorphization and roughening of the surface under the pillars, as shown in the inset of Figure 5(b). Thus, the observed reduction in thermal conductivity was attributed to the surface roughness and the amorphous layer under the pillars. Indeed, amorphous layers at the surfaces are known to reduce the thermal conductivity of nanostructures [61,64–66] and can diffusely scatter phonons stronger than just surface roughness [67].

Thus, the effect of the thermal conductivity reduction by the phonon interference in pillars could not be verified experimentally yet and remains a purely theoretical prediction. However, the experimentally observed 20% reduction in thermal conductivity is interesting to compare with the reduction obtained in other silicon-based nanostructures. On one hand, a stronger reduction was measured in silicon with pores (>50%) [9,18,19], nanodots (>70%) [13,68], polycrystalline grains (>80%) [15,69,70], dopants (>50%) [71,72] or germanium atoms (>70%) [14,71,73]. On the other hand, the 20% reduction by the pillars [63] is comparable to the reduction by holes (20–25%) [21,74] or slits (20–30%) [75] covering the same relative area. This relative comparison shows that pillars could achieve the same reduction in the thermal conductivity without sacrificing the material volume or introducing scattering points inside the bulk of the material.

## 6. Summary and outlook

We reviewed the past decade of studies on pillar-based PnC. The pioneering works mainly studied how local resonances flatten phonon dispersion and open phononic bandgaps, which can suppress propagation of phonons at gigahertz frequencies. In the past few years, this idea expanded beyond the phononic bandgaps and researchers began dreaming about the suppression of phonons in the terahertz frequency range thus reducing the thermal conductivity of materials [30,32,33,55]. However, the Brillouin light scattering experiments could confirm changes in phonon dispersion only up to a few tens of gigahertz [31,48], which affects only a negligible part of the thermal phonon spectrum at room temperature.

Nevertheless, many simulations obtained the reduced thermal conductivity in pillar-based PnCs. The origin of this reduction remains somewhat unclear because the thermal conductivity could be reduced by the local resonances in pillars, the periodicity of pillars, the diffuse scattering on pillars, or by some of these mechanisms together. Molecular dynamics simulations cannot easily distinguish impacts of these mechanisms and are limited to the structures of a few nanometers in size, whereas FEM simulations can study structures of any size but do not take diffuse scattering into account. Future simulations should try to isolate each of these mechanisms by studying, for instance, disordered arrays of pillars, pillars with no local resonances in the relevant frequency range or comparing the results of elastic (without diffuse scattering) and non-elastic simulation models.

The predicted reduction in thermal conductivity might be promising for silicon-based thermoelectric devices because pillar-based PnCs can presumably keep high electrical conductivity so that the thermal conductivity reduction might directly enhance the thermoelectric efficiency. Also, pillar-based PnCs do not require to sacrifice the material volume and thus avoid weakening of the mechanical strength and the power generation. Yet, the experiments could not demonstrate that pillars can reduce the thermal conductivity by phonon interference, let alone increase thermoelectric performance. Future experimental studies should strive for miniaturization and create monolithic PnCs to exclude the phonon scattering at the interface. This can be achieved either by the top-down etching or by the bottom-up nanowires growth. Moreover, as phonons can lose their coherence upon surface scattering, experimentalists should either minimize the surface roughness or perform the measurements at low temperatures, at which the phonon wavelengths are longer than the surface roughness. Finally, to demonstrate the potential for thermoelectric applications, experiments should confirm that pillars leave the electrical conductivity and Seebeck coefficient unaffected.

Thus, the phonon and heat conduction in pillar-based PnCs remains a field of theoretical debates, experimental challenges, and promising routes for future thermoelectrics.

## Note

1. At least in simulations that used finite element method or lattice dynamics and did not consider diffuse scattering processes.
